# Metal Exposure in Child Workers: Assessing Hazards in Surgical Instrument Manufacturing Workshops

**DOI:** 10.1289/ehp.120-a403b

**Published:** 2012-10-01

**Authors:** Tanya Tillett

**Affiliations:** Tanya Tillett, MA, of Durham, NC, is a staff writer/editor for *EHP*. She has been on the *EHP* staff since 2000 and has represented the journal at national and international conferences.

The International Labor Organization estimates that tens of millions of children work in hazardous conditions around the world. In Sialkot, Pakistan, the primary site for surgical instrument production in the developing world, many children labor in dusty workshops producing these items. In a new cross-sectional study researchers report high metal exposures possibly linked to higher rates of respiratory ailments and oxidative DNA damage among children working in 21 Sialkot workshops [*EHP* 120(10):1469–1474; Sughis et al.].

The authors surveyed 104 working children and 75 nonworking schoolchildren aged 10–14 to assess working conditions, exposures to tobacco smoke (active and passive) and to biomass smoke, use of medications, and social class. Urine samples from each participant were analyzed for 20 metals. For 145 children the authors also measured urinary 8-hydroxydeoxyguanosine (8-OHdG), a DNA breakdown product that serves as a biomarker of oxidative stress. They assessed lung function with a pocket spirometer and calculated the average of five consecutive blood pressure readings to classify each child’s blood pressure as normal or prehypertensive.

Most of the working children spent 6 days a week in workshops that typically had poor ventilation and lighting, some for up to 12 hours a day. The children mainly ground and polished instruments, and none of them used personal protective equipment.

**Figure:**
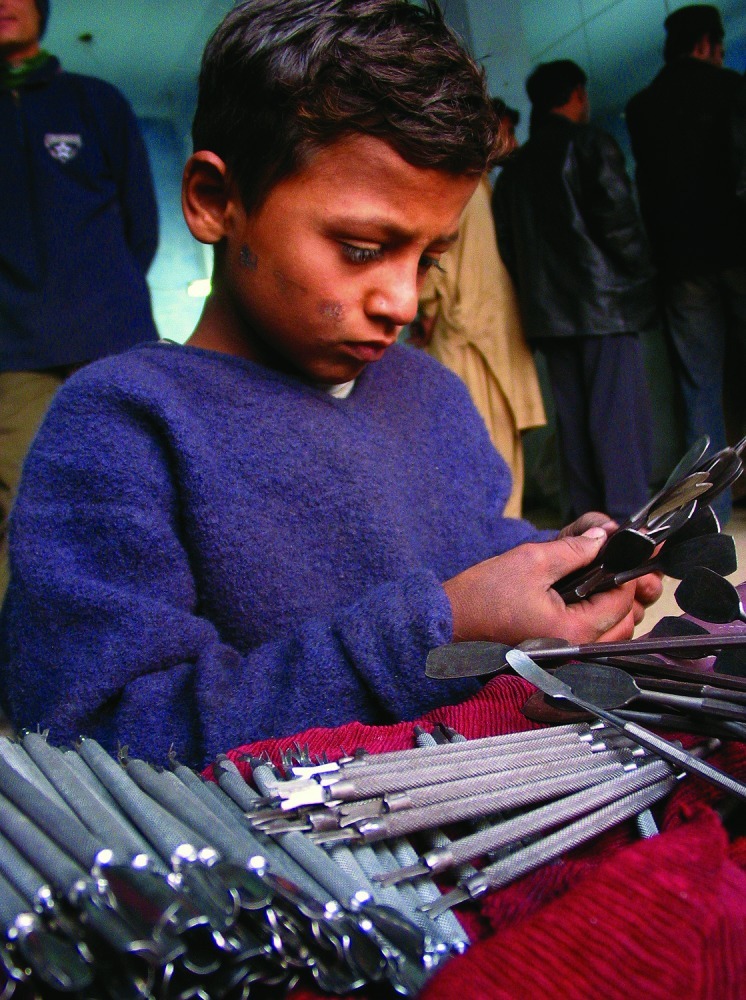
Twelve-year-old Kabir Qadeer sorts surgery instruments at a workshop in Sialkot, Pakistan. © AP Photo/B.K.Bangash

Compared with the schoolchildren, the working children had higher average urinary concentrations of several steel-related metals. Most notably, chromium levels were 35 times higher in the working children, with values usually exceeding the adult limit of 25 µg/L set by the American Conference of Governmental Industrial Hygienists. Urinary 8-OHdG concentrations did not differ significantly between working children and schoolchildren, although 8-OHdG was significantly correlated with urinary nickel and with overall metal exposure.

The working children reported more respiratory symptoms and asthma than the schoolchildren, but their pulmonary function values were significantly better. The authors suggest several possible explanations, such as the fact that acceptable spirometry results were available for only 37% of the working children compared with 90% of the schoolchildren. No significant differences were seen in blood pressure between the two groups.

The study’s strengths include individual biomonitoring of the working children’s exposure to metals. Limitations include potential bias in recruitment and difficulty in assessing respiratory end points. The authors write that child labor is a complex issue and that poor parents should not be stigmatized for sending their children to work. Despite a lack of serious health effects observed at the time of study, the findings do present evidence that children may suffer occupational illness in the future, even if they discontinue this work. It is therefore important to implement and enforce measures to reduce hazardous working conditions.

